# In vitro anti-HIV activity of crude extracts from *Tinospora cordifolia*

**DOI:** 10.1186/1471-2334-12-S1-P10

**Published:** 2012-05-04

**Authors:** Mamidala Estari, Lunavath Venkanna, Annem Srinivas Reddy

**Affiliations:** 1 Infectious Diseases Research Lab, Department of Zoology, Kakatiya University, Warangal, Andhra Pradesh, India

## Background

Human immunodeficiency virus (HIV) infection causes acquired immune deficiency syndrome (AIDS) and is a global public health issue. Anti-HIV therapy involving chemical drugs has improved the life quality of HIV/AIDS patients. However, emergence of HIV drug resistance, side effects and the necessity for long-term anti-HIV treatment are the main reasons for failure of anti-HIV therapy. Therefore, it is essential to isolate novel anti-HIV therapeutics from natural resources. The aim of the present study was to evaluate the invitro anti-HIV activity of *T. cordifolia* plant extracts.

## Methods

Extracts were prepared from dried leaves in n-hexane, dichloromethane, ethyl acetate and n-butanol. A toxicity study was performed on all crude extracts using peripheral mononuclear blood cells (PBMCs) isolated from whole blood. HIV-1 RT inhibition activity of the all solvent extracts of *T. cardifolia* was determined using a commercial kit.

## Results

Among the tested extracts, the n-hexane and n-butanol crude extracts of *T. cordifolia* showed moderate cytotoxic activities against PBMCs with CC50 values ranging from 5.7-12.0 µg/ml. In the HIV-1 reverse transcriptase assay *T. cardifolia* plant extracts showed good inhibitory activity, which was near that of the reference drug. Ethyl acetate extract shows 85 percentage of HIV-1 RT inhibition activity at a concentration of 20 mg/ml.

## Conclusion

The leaves of *T. cardifolia* extracts are shows anti-HIV 1 activity and this plant has great potential for developing useful drugs. Extraction of important biologically-active phytochemicals from this plant will certainly be helpful in protecting and treating various viral diseases in human beings.

